# Incidental Right Aortic Arch in a 55-Year-Old Male With Cerebral Palsy: A Radiologic Pitfall Mimicking Pulmonary Pathology

**DOI:** 10.7759/cureus.115153

**Published:** 2026-08-25

**Authors:** Mercedes Malone, Carolina C Ferraz, Tarek Zaho

**Affiliations:** 1 Internal Medicine, HCA Florida North Florida Hospital, Gainesville, USA; 2 Medicine, Universidade Santo Amaro School of Medicine, Sao Paulo, BRA; 3 Regenerative Medicine, Wake Forest Institute of Regenerative Medicine, Winston-Salem, USA

**Keywords:** congenital vascular anomaly, kommerell diverticulum, pulmonary mass mimic, radiologic pitfall, right aortic arch

## Abstract

We present the case of a 55-year-old male with a past medical history of cerebral palsy, obstructive sleep apnea (OSA), seizures, and generalized anxiety disorder who presented with recurrent episodes of respiratory distress and was incidentally found to have a right aortic arch (RAA) on radiologic imaging. Although this anatomical variation is rare, this case serves as a teaching point to raise awareness of the existence of a RAA and how it can be mistaken for a lung lesion, mass, or malignancy. Studies have shown that this anatomical abnormality can also give rise to subtle esophageal and respiratory symptoms. In addition, some patients diagnosed with RAA, especially older adults, have been found to have concurrent vascular anomalies, such as right coronary artery stenosis, left and/or right subclavian artery stenosis, and aortic dissection.

## Introduction

The aortic knob is described as a hump-like appearance of the aortic arch and is typically most apparent on radiologic imaging. In most individuals, the aortic arch is found on the left side of the heart. However, the aortic arch can be appreciated on the right side in around 0.05% to 0.1% of the general population [1]. Embryologic processes frequently determine the laterality of the aortic arch. Therefore, abnormal embryologic development can lead to a congenital variation from what is considered typical and more predominant. A right-sided aortic arch (RSAA) was detected in 0.6% of all fetal cardiac examinations and in 5.0% of all cardiac abnormalities [2].

Although often benign, variations in aortic arch anatomy may pose diagnostic challenges and, in selected cases, carry important clinical implications due to compression of adjacent structures or misinterpretation as mediastinal pathology on thoracic imaging [3].

The aortic head and neck structures are primarily developed from six pairs of pharyngeal arches. Initially, the arches are arranged in pairs but become asymmetrical after remodeling [4]. The fourth pharyngeal arch predominantly forms the aortic arch. The laterality of the aortic arch is defined by the pathway taken relative to the bronchus. Of the fourth pharyngeal arches, the fourth right arch usually regresses, and the left one gives rise to the normal aortic arch, which arches to the left of the main bronchus. However, on rare occasions, the left arch paradoxically regresses [5]. Thus, the final arch arises from the right pharyngeal arch, leading to a RSAA that passes to the right of the bronchus.

There are three main subtypes of RSAA according to Edwards’ model. Type I, which represents 59% of all RSAAs, is associated with congenital heart diseases such as truncus arteriosus or tetralogy of Fallot. Type II is formed by an aberrant left subclavian artery arising from a diverticulum of Kommerell as a fourth branch from the aorta, representing 40% of RSAAs and is not associated with congenital heart diseases. Finally, Type III is caused by obliteration of the left subclavian artery with collateralization, representing 1.5% of all RSAAs [5]. If the right aortic arch (RAA) is a counterpart of the standard left-sided aortic arch, it does not pose critical physiologic abnormalities. Although mostly asymptomatic, there are some situations in which it can be related to cases of intractable exertional dyspnea [6].

Although adult cases of RAA with an aberrant left subclavian artery and Kommerell diverticulum have previously been described, the present case illustrates a clinically important diagnostic intersection. The anomaly was identified in an adult with obstructive sleep apnea (OSA), cerebral palsy, recurrent respiratory illnesses, and severe acute hypoxic and hypercapnic respiratory failure. The case highlights both the potential for an abnormal right mediastinal vascular contour to mimic thoracic pathology and the difficulty in determining whether a congenital vascular anomaly is incidental or contributes to reduced airway reserve in the presence of substantial competing respiratory factors.

## Case presentation

A 55-year-old male with a past medical history of cerebral palsy, OSA, seizures, and generalized anxiety disorder presented to our emergency department with chief complaints of nasal congestion, cough, and shortness of breath for two days prior to admission. His vital signs were as follows: blood pressure, 118/76 mmHg; pulse, 83 beats/min; respiratory rate, 19 breaths/min; and temperature, 36.9°C. He reported gradual worsening since symptom onset.

**Figure 1:**
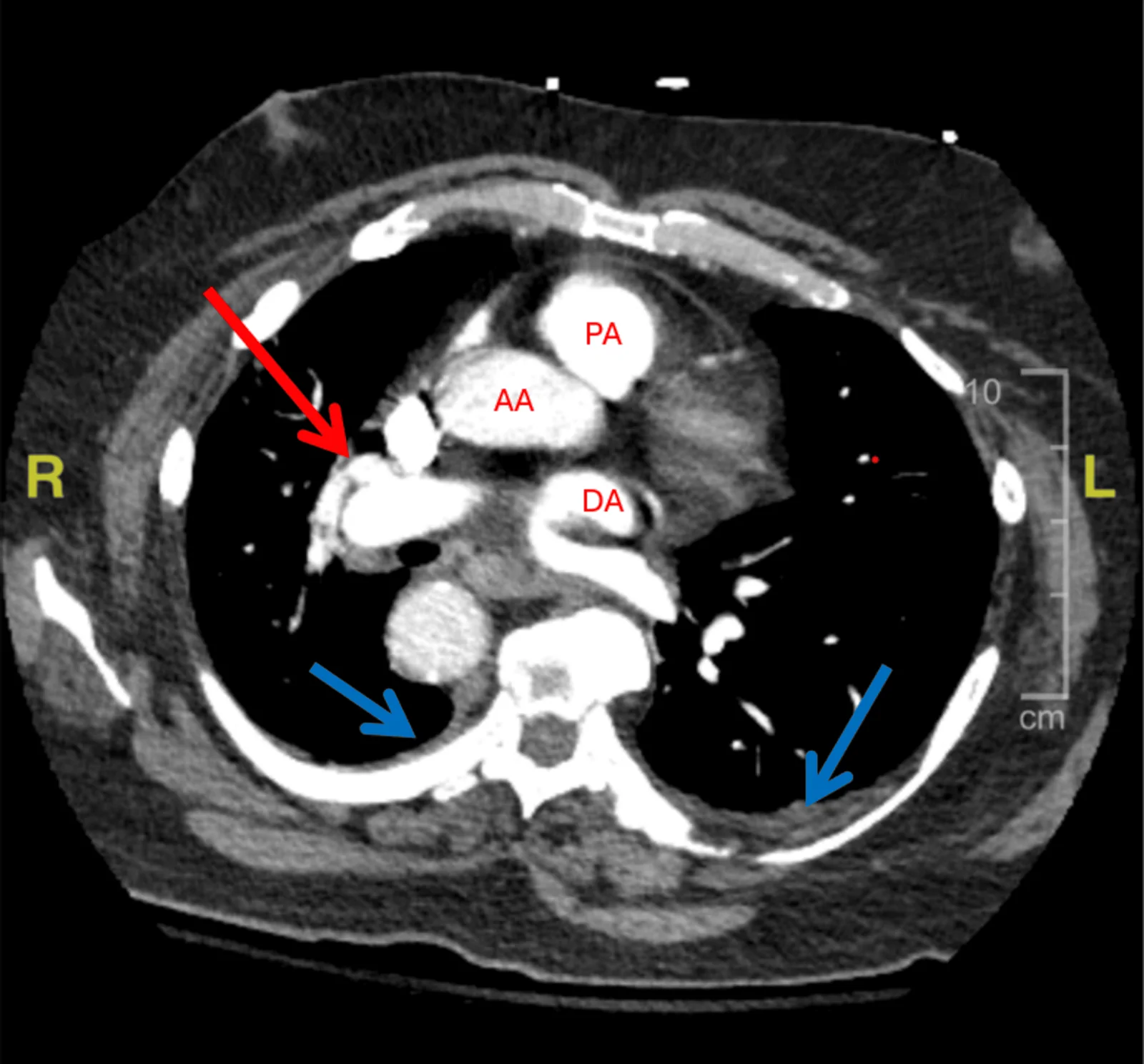
CTA of the neck There are moderate-sized bilateral pleural effusions (blue arrows) and bilateral airspace opacities. The Kommerell diverticulum measures approximately 4.0 cm (red arrow), a size associated with an increased risk of compressive symptoms and warranting vascular surgery evaluation. CTA: computed tomography angiography; AA: abdominal aorta; PA: pulmonary artery; DA: descending aorta; R: right; L: left

He also had a recent hospitalization nearly one year earlier, during which a stroke was considered a possible diagnosis after the patient lost consciousness and became cyanotic while at work. The stroke workup was negative, and his presentation was deemed likely related to a seizure. During that admission, computed tomography angiography (CTA) of the neck revealed an incidental RSAA (Figure 1). At that time, he also had diminutive bilateral pleural effusions and had recently recovered from coronavirus disease 2019 (COVID-19) pneumonia and influenza virus infection. He was treated for seizures and discharged home with recommendations for follow-up with a neurologist.

A retropharyngeal course of both internal carotid arteries was appreciated. No flow-limiting carotid stenosis was identified. Additionally, no flow-limiting vertebrobasilar stenosis was identified. During this latest hospitalization, he was found to have significant hypoxia upon admission, with oxygen saturation as low as 82% on room air. He was placed on three liters of oxygen administered through a nasal cannula, and his oxygen saturation improved to 99%.

The acute respiratory failure in this patient was most plausibly multifactorial. The temporal onset of cough and dyspnea, pulmonary airspace disease, leukocytosis, elevated lactate, and clinical response to antimicrobial and supportive therapy support infectious pneumonia as the principal precipitating factor. OSA may also have increased susceptibility to hypoventilation and carbon dioxide retention during acute illness.

An RAA with an aberrant left subclavian artery and Kommerell diverticulum can form a vascular ring and, when sufficiently enlarged or unfavorably positioned, may compress the trachea or esophagus. However, the available imaging in this patient did not provide quantitative evidence of clinically significant tracheal narrowing, and no bronchoscopy, dynamic airway imaging, or pulmonary function assessment was available to demonstrate fixed or dynamic obstruction. The vascular anomaly should therefore be considered a possible contributor to reduced airway reserve rather than a proven cause of the acute hypoxic and hypercapnic respiratory failure.

**Figure 2:**
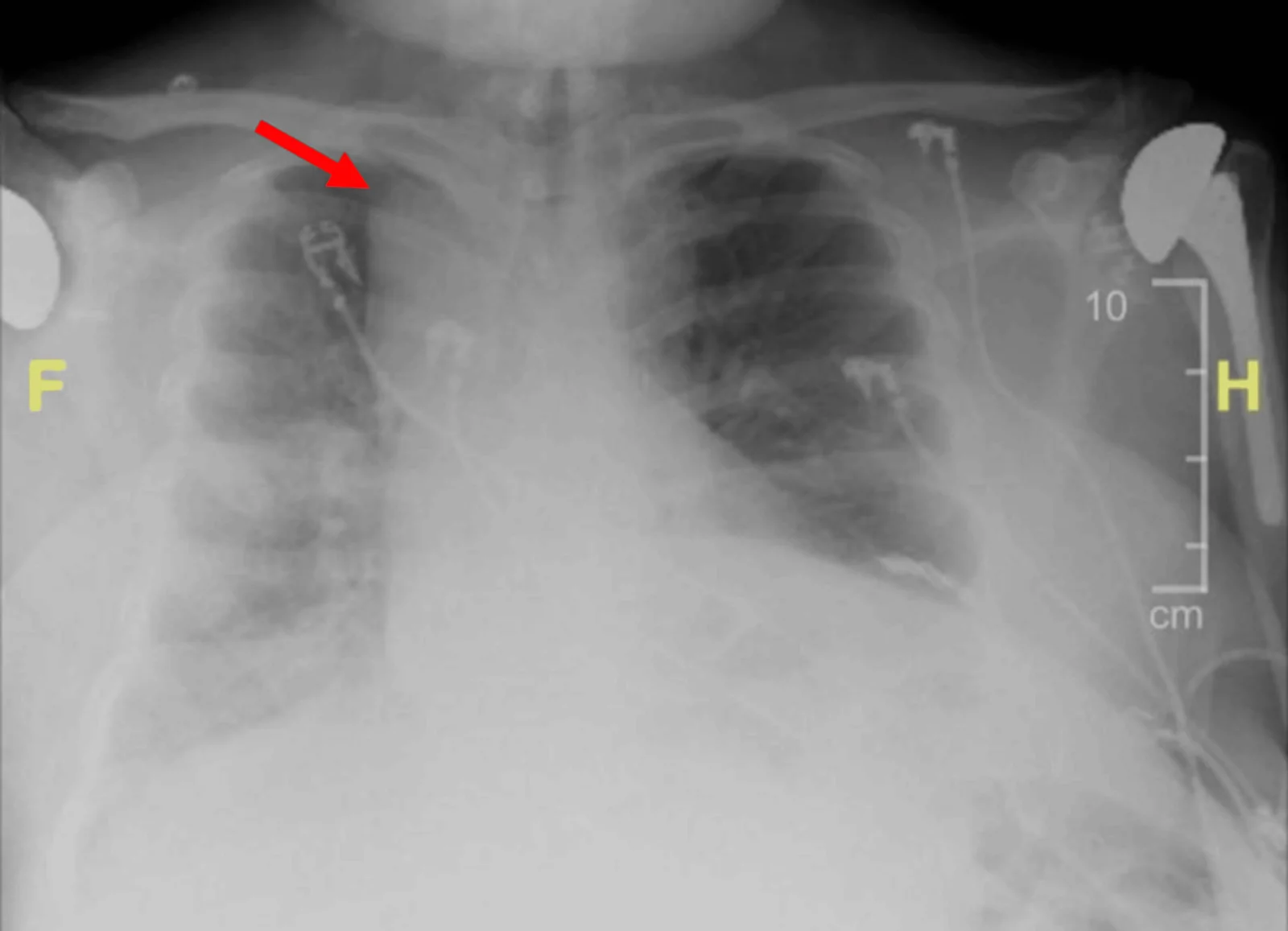
Chest X-ray The chest X-ray demonstrates a right-sided aortic knob, an important radiographic finding that may be misinterpreted as mediastinal pathology. F: field; H: hila

A CT scan did not demonstrate any evidence of pulmonary embolism, although a chest X-ray revealed airspace disease in the right mid and lower lung zones. The chest X-ray also demonstrated the same right-sided aortic knob that was noted on the prior CTA scan of the neck (Figure 2). The patient was admitted to the hospital for further management of acute hypoxic respiratory failure due to infectious pneumonia, which required additional investigations.

On the second day of admission, the patient developed worsening tachypnea, altered mental status, and increased work of breathing. Oxygen saturation declined to approximately 80% despite six liters of supplemental oxygen via nasal cannula. Noninvasive ventilation with bilevel positive airway pressure (BiPAP) was initiated. However, the patient continued to deteriorate with persistent hypercapnia and declining mental status. Given the failure of noninvasive support, the decision was made to proceed with endotracheal intubation and mechanical ventilation. Arterial blood gas analysis at that time revealed a pH of 7.25 with a PaCO₂ of 74 mmHg, consistent with acute hypercapnic and hypoxic respiratory failure. A transthoracic echocardiogram (TTE) was also performed and showed findings similar to those observed on the previous TTE from his hospitalization one year earlier.

Sputum and blood cultures were collected, and testing for urinary Legionella and *Streptococcus pneumoniae* was performed within the first day of admission. Ultimately, all results were negative. Testing for COVID-19 and influenza A and B was also negative.

After completing antibiotic therapy and receiving supplemental oxygen during hospitalization, his condition improved significantly. He was evaluated for home supplemental oxygen and met the qualification criteria. He was discharged with recommendations for outpatient vascular surgery and pulmonology follow-up. However, he was subsequently lost to follow-up despite multiple attempts at contact. Although his acute respiratory failure was attributed primarily to infectious pneumonia, the presence of a RSAA with a Kommerell diverticulum may have contributed to reduced airway reserve.

## Discussion

An RSAA is a rare congenital anomaly, occurring in 0.05% to 0.1% of radiologic series and 0.04% to 0.1% of autopsy series [3,4,7]. This anatomic anomaly is typically discovered incidentally on radiologic imaging performed to investigate other potential cardiopulmonary pathology. In the case of our patient, it was not known that he had an RAA until he underwent CTA of the neck for suspected stroke-like symptoms. Although usually benign, an RSAA can be mistaken for another entity, such as a lung or mediastinal mass, by the untrained eye. It is important to keep this possibility in mind when an abnormally positioned aortic knob is identified.

Although an RSAA with an aberrant left subclavian artery is often asymptomatic, the anatomic configuration can result in clinically significant airway compromise through the formation of a partial or complete vascular ring. In such cases, posterior displacement or encirclement of the trachea may lead to extrinsic tracheal compression, reducing airway caliber and compliance [8].

This mechanical limitation impairs effective ventilation, particularly during periods of increased respiratory demand or superimposed pulmonary infection, and may contribute to the development of hypercapnic respiratory failure. In our patient, the combination of acute infectious pneumonia, underlying OSA, and possible tracheal compression from the malpositioned aortic arch likely resulted in reduced ventilatory reserve, potentially amplifying the severity of respiratory compromise in the setting of acute illness [9,10].

Although usually asymptomatic, there have been reports of this congenital anomaly contributing to symptoms such as chronic cough, dyspnea, exertional asthma, and dysphagia [6,11]. The mechanism underlying these symptoms relates to the formation of a vascular ring that impinges on nearby anatomic structures, including the trachea and esophagus [6].

The patient we present in this case had multiple overlapping conditions that could have contributed to his respiratory insufficiency. He was diagnosed with OSA by polysomnography and was compliant with continuous positive airway pressure (CPAP) therapy. He also had a history of COVID-19 and influenza infection, OSA, and recent-onset upper and lower respiratory tract infection symptoms.

RAA has been associated with rare conditions such as tetralogy of Fallot, truncus arteriosus, and genetic abnormalities such as DiGeorge syndrome (22q11 deletion) [6,7]. No established association between cerebral palsy and an RSAA has been reported in the literature. However, this warrants further investigation in future studies.

Severe respiratory insufficiency in this patient is more appropriately attributed to acute infectious pneumonia and underlying comorbidities, with the vascular anomaly representing a potential contributing anatomic factor [5,6].

Despite the rarity of this condition, it is important to emphasize that patients diagnosed with RAA should be attentively examined for other concurrent vascular abnormalities. Older adults represent a particularly high-risk population because of age-related cardiovascular changes. Involvement of the large arteries and the vertebrobasilar system is of particular concern. With advancing age, aortic diameter and arch length increase, and the aorta becomes more tortuous [8-10]. Arazińska et al. [3] reported concomitant vascular anomalies, including right carotid artery stenosis, right subclavian artery stenosis, left subclavian artery stenosis, and type B aortic dissection, in four of 11 patients with type II RSAA [11].

Management of Kommerell diverticulum depends on symptoms, anatomy, diverticular dimensions, interval growth, associated aortic pathology, patient comorbidities, and operative risk. The 2022 American College of Cardiology (ACC)/American Heart Association (AHA) aortic disease guideline states that repair may be reasonable when the diverticular orifice exceeds 3.0 cm, the combined diameter of the diverticulum and adjacent descending aorta exceeds 5.0 cm, or both, after consideration of individual anatomy and comorbidities. The reported 4.0-cm measurement in this patient therefore required confirmation using standardized thoracic aortic measurements because it was not initially specified whether the value represented the diverticular orifice, maximal sac diameter, or combined aortic dimension.

Available treatment strategies include open repair with diverticular resection and reconstruction of the aberrant subclavian artery, hybrid repair combining supra-aortic or subclavian revascularization with thoracic endovascular aortic repair, and selected total endovascular approaches. No single technique is appropriate for all patients. Open repair may offer definitive exclusion and decompression but carries a greater physiologic burden, whereas hybrid and endovascular strategies may reduce operative invasiveness but depend on adequate landing zones, arch anatomy, and long-term device surveillance. Contemporary reviews support individualized treatment selection according to clinical presentation, anatomy, age, comorbidity, and institutional expertise. Patients managed nonoperatively require clinical review and serial CTA or magnetic resonance angiography (MRA) to monitor symptoms, diverticular dimensions, and interval growth, although universally accepted Kommerell-specific surveillance intervals have not been established [12-15].

In this case, the patient's respiratory failure was likely multifactorial rather than solely attributable to the RSAA or associated vascular anomaly. Acute infectious pneumonia was the most direct precipitating factor, and the patient's underlying OSA and recent respiratory infections may have further reduced his respiratory reserve. Although the vascular anomaly may have contributed anatomically through possible airway compression, the patient's clinical improvement without definitive surgical correction suggests that the anomaly was unlikely to be the primary independent cause of respiratory failure. Instead, it may have acted as an additional predisposing or exacerbating factor in the setting of acute pulmonary illness.

Limitations

This case report has several limitations. First, as a single-patient report, it cannot establish a causal relationship between the RSAA, the aberrant vascular anatomy, and the patient's respiratory failure. The patient had multiple concurrent conditions that may have contributed to his respiratory compromise, including acute infectious pneumonia, OSA, recent respiratory infections, and underlying neurologic comorbidity. Therefore, the vascular anomaly should be interpreted as a potential contributing anatomic factor rather than the sole cause of his presentation.

Second, although imaging demonstrated an RSAA and associated vascular abnormality, the degree of dynamic airway compression was not directly assessed with dedicated airway imaging, bronchoscopy, or pulmonary function testing. As a result, the precise functional impact of the vascular ring or Kommerell diverticulum on airway caliber and ventilation could not be fully determined.

Third, long-term clinical follow-up and serial imaging data were limited. Such follow-up would be useful for evaluating progression of the Kommerell diverticulum, development of compressive symptoms, and the need for vascular surgical intervention. Finally, because an RSAA is rare and often discovered incidentally, further studies and larger case series are needed to better define its clinical significance, natural history, and optimal management in adults with overlapping cardiopulmonary conditions.

Fourth, heart failure with preserved ejection fraction should also be considered a potential contributor to hypoxemia and respiratory compromise in similar clinical presentations. However, N-terminal pro-B-type natriuretic peptide (NT-proBNP) was not obtained during this hospitalization, which limits our ability to assess this possibility. Therefore, heart failure with preserved ejection fraction (HFpEF) cannot be definitively excluded as a contributing factor in this case.

## Conclusions

RAA is an incidental finding that is most often discovered during radiologic evaluation and is typically asymptomatic in most patients. To the untrained eye and without a high index of suspicion, RAA may be mistaken for a lung or mediastinal mass or abscess, especially in a patient with recurrent respiratory symptoms. Additionally, the presence of RAA can contribute to the impingement of nearby anatomical structures and the development of dyspnea. Our report aims to shed light on this congenital anomaly, which, although rare, requires careful evaluation and management because of its potential for severe complications and associated vascular abnormalities.
